# Preferential allele amplification leading to RyR1 misgenotyping in a malignant hyperthermia susceptible individual

**DOI:** 10.1186/1471-2253-14-S1-A16

**Published:** 2014-08-18

**Authors:** Asensio Gonzalez, Martine Singer, Christoph Noppen, Albert Urwyler, Thierry Girard

**Affiliations:** 1 Departments of Research and Anesthesiology, University Hospital Basel, 4031 Basel, Switzerland; 2 Genetik/Molekularbiologie, Viollier AG, 4123 Allschwil, Switzerland

## Background

Many current methods for the detection of gene variants relevant for inherited disorders like malignant hyperthermia (MH) rely on the polymerase chain reaction (PCR). A positive PCR result for the genetic region of interest is required for downstream analysis like sequencing, restriction digestion or probe hybridization. However, it is sometimes overlooked that in order to obtain a true genotype, both paternal and maternal alleles must be represented in the PCR product. We present the case of a preferential PCR amplification of one ryanodine receptor subtype 1 (RyR1) mutant allele that led to apparent homozygosity of a proband.

## Material and methods

A woman, 23 y.o. with family history of MH susceptibility was initially genotyped as homozygous carrier of the MH causative RyR1 mutation 2434Gly>Arg. This unusual genotype was investigated by further PCR and sequencing.

## Results

Sequencing of the genomic region from the biological parents of the proband revealed that only the father was a mutation carrier. This result was confirmed by using an additional, extended PCR that covered the target exon plus adjacent introns, casting doubt on the initial genotype call of the daughter. Using the same extended PCR in the daughter, indicated that she indeed carried a wild-type along with the mutated RyR1 allele. We endorsed this genotype over the initial one because the presence of additional intronic polymorphisms (SNPs) provided evidence that both parental alleles were represented in the extended amplicon. Moreover, taking advantage of the Inherited Disease Panel and a next generation sequencing platform (Ion Torrent, Personal Genome Machine, Life Technologies), we could confirm the heterozygosity for the RyR1 mutation by a completely different method.

## Conclusions

These results highlight that PCR amplification bias of one parental allele may completely mask the presence of the second parental allele, giving raise to apparent, but false, homozygosity. An appropriate quality control to establish the diploid nature of the material to be sequenced is thus important for accurate PCR-based genotyping, particularly for the diagnosis of autosomal dominant disorders like MH. For this purpose, we suggest the use of extended regions reaching neighboring SNPs.

